# Rescue balloon-assisted remodeling technique for protrusion of coil loop: A case report

**DOI:** 10.1097/MD.0000000000025783

**Published:** 2021-05-14

**Authors:** Hak Cheol Ko

**Affiliations:** a Department of Neurosurgery, Stroke and Neurological Disorders Centre, Kyung Hee University Hospital at Gangdong; b College of Medicine, Kyung Hee University, Seoul, Korea.

**Keywords:** balloon-assisted remodeling, coil protrusion, endovascular embolization, intracranial aneurysm

## Abstract

**Rationale:**

Among the possible complications during endovascular embolization of intracranial aneurysms, coil protrusion into the parent artery is associated with parent artery occlusion or thromboembolic of the distal arteries. There is no clearly established management strategy for coil protrusion. This report demonstrates our experience with balloon-assisted remodeling to reposition a protruded coil loop.

**Patient concerns:**

A 53-year-old man was admitted to our hospital with severe bursting headache, nausea, and vomiting. Computed tomography showed subarachnoid hemorrhage and digital subtraction angiography revealed an anterior communicating artery aneurysm. We decided to obliterate the aneurysm with endovascular embolization using detachable coils

**Diagnosis:**

A small loop protruded into the parent artery during the removal of the microcatheter.

**Interventions:**

We performed successful repositioning of the protruded coil loop using balloon inflation.

**Conclusion:**

The rescue balloon-assisted remodeling technique was useful in the management of protrusion of a small coil loop into the parent artery during endovascular coil embolization of an intracranial aneurysm. The procedure was associated with minimal complications.

## 1 Introduction

Endovascular embolization using a detachable coil system for the management of intracranial aneurysms has shown rapid technical developments since its inception.^[1–3]^ Its safety and effectiveness are proven to be non-inferior to those of microsurgical clipping.^[4,5]^ Although it has several benefits, possible intraprocedural complications such as coil protrusion into the parent artery, thromboembolic event, and aneurysm rupture are still encountered. Among these complications, coil protrusion into the parent artery might lead to parent artery occlusion or thromboembolism of the distal artery that can result in catastrophic ischemic stroke.^[6–8]^ The reported incidence of coil protrusion is 2% to 6% and it has not reduced over the years.^[6–8]^ There are no clearly established management strategies for coil protrusion.^[2]^ Based on our experience, the balloon-assisted remodeling technique for the treatment of coil protrusion into the parent artery provides a possible solution and decreases the use of additional devices such as snares and retriever devices.

## 2 Case description

**Figure 1:**
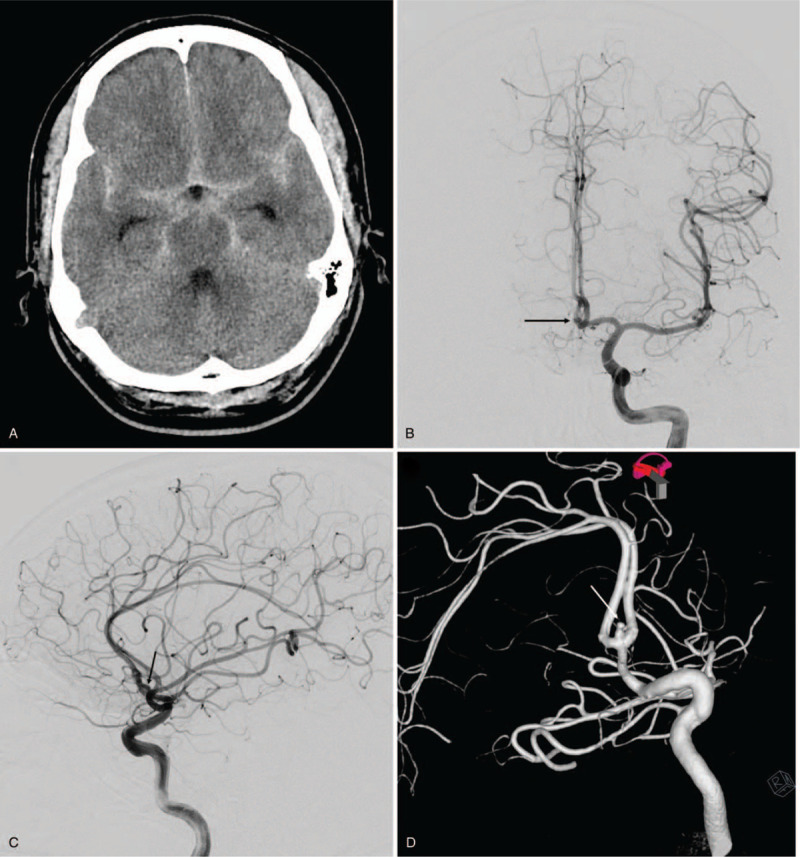
(A) Axial view of non-enhanced computed tomography showing subarachnoid hemorrhage. (B and C) Anteroposterior and lateral view of left internal carotid angiogram showing an aneurysm originating at the anterior communicating artery (black arrow). (D) Volumetric 3-dimensional reconstruction of digital subtraction angiography showing a snowman-like shaped aneurysm on working projection (white arrow).

A 53-year-old man presented to our emergency room with severe bursting headache, nausea, and vomiting. He had a clear state of consciousness without any other neurological deficits and exhibited a score of 15 on the Glasgow Coma Scale (Hunt and Hess grade II). Computed tomography indicated subarachnoid hemorrhage at the suprasellar cistern, interhemispheric fissure, and bilateral sylvian fissure (Fisher grade III) (Fig. 1A). Digital subtraction angiography (DSA) revealed an aneurysm originating at the anterior communicating artery (Fig. 1B and C). The aneurysm had a snowman-like shape with a neck diameter of 2.45 mm and a height of 2.95 mm (Fig. 1D). Endovascular coil embolization was considered a relevant strategy to manage the aneurysm. Under general anesthesia, a 6 Fr (1.80 mm) guide catheter (Asahi Fubuki, Asahi Intecc Co., Ltd) was inserted through the right femoral artery and located at the petrous internal carotid artery. To determine the optimal working projection of the aneurysm, DSA volumetric three-dimensional reconstructions were performed (Fig. 1D). Based on the working projection, a microcatheter (Excelsior SL-10 PreShaped 90 2-tip marker, Stryker, Kalamazoo, MI, USA) was introduced into the aneurysmal sac along the microwire (Transend, Stryker, Kalamazoo, MI, USA) (Fig. 2 A). Subsequently, a bolus dose of unfractionated heparin (3000 IU) was administered intravenously. Three detachable coils were placed inside the aneurysm in regular sequence (2 GALAXY G3^TM^ MINI Microcoil sized 2 mm × 3 cm [Johnson & Johnson, New Brunswick, NJ, USA] and a Target Detachable Coil sized 1 mm × 2 cm, [Stryker, Kalamazoo, MI, USA]). Control angiogram showed obliteration of the aneurysm with good patency of the parent artery (Fig. 2 B). Therefore, after detaching the last coil, we tried to remove the microcatheter carefully. During this maneuver, the coil loop of the last coil protruded into the parent artery wall (Fig. 2 C). Aggrastat (Tirofiban hydrochloride) was prepared for treating possible thrombus formation around the protruding coil loop. A thrombus was observed in the 10-minute delayed angiogram. There was a high possibility that the thrombus would continue to develop as long as the coil loop remained protruded into the parent artery (Fig. 2 D). Hence, we attempted to remodel the protruded coil loop using a balloon instead of Aggrastat. A Scepter XC^TM^ balloon catheter (MicroVention^TM^, Inc.; Aliso Viejo, CA, USA) was placed along the protruded coil loop and the aneurysm neck, followed by slow inflation under fluoroscopy (Fig. 3A, see Supplemental Video {Supplemental Video. Video that demonstrates the rescue balloon-assisted remodeling technique for coil repositioning, 5 seconds, 2.62MB}). The protruded coil loop was pushed back into the aneurysmal sac successfully (see Supplemental Video), resulting in the disappearance of the thrombus (Fig. 3B). Final angiogram demonstrated that the aneurysm was well obliterated without coil prolapse into the parent artery or thromboembolic events. The patient was discharged without any neurologic abnormalities. A 6-month follow-up magnetic resonance angiogram showed that there was no recurrence of the aneurysm and the flow of the parent vessel was well maintained.

**Figure 2:**
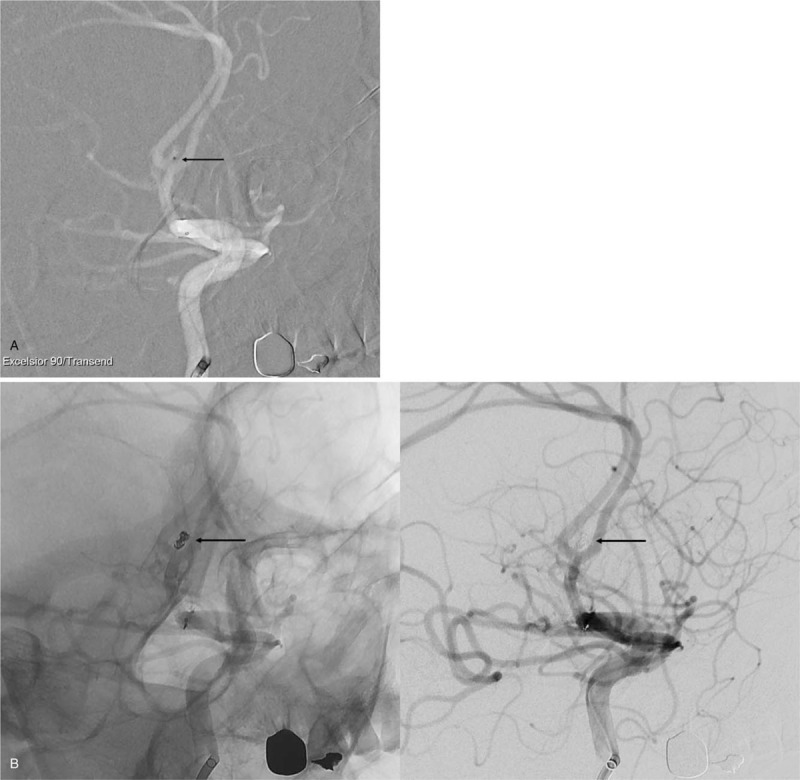
Intraprocedural working projection view of digital subtraction angiography. (A) A microcatheter in the aneurysmal sac (black arrow). (B) Obliteration of aneurysm with 3 detachable coils with good patency of the parent artery (black arrow). (C) Coil protrusion into the parent artery during removal of microcatheter (black arrow). (D) Thrombus formation around the protruding coil (black arrow).

**Figure 2(Continued):**
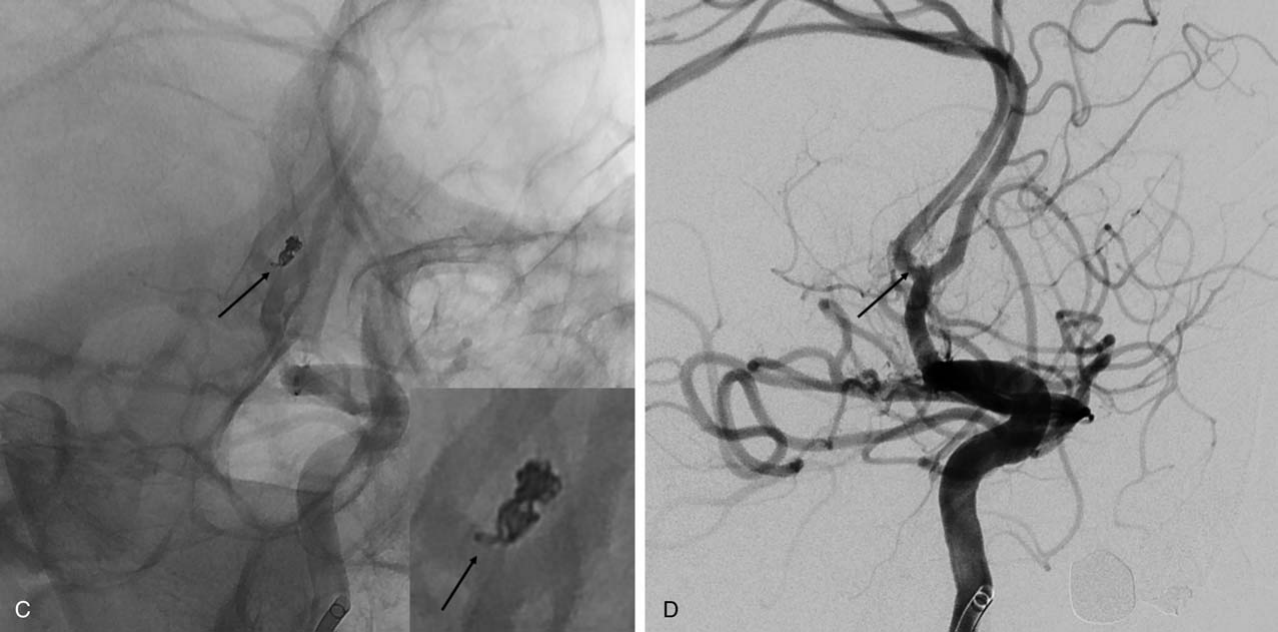
Intraprocedural working projection view of digital subtraction angiography. (A) A microcatheter in the aneurysmal sac (black arrow). (B) Obliteration of aneurysm with 3 detachable coils with good patency of the parent artery (black arrow). (C) Coil protrusion into the parent artery during removal of microcatheter (black arrow). (D) Thrombus formation around the protruding coil (black arrow).

**Figure 3:**
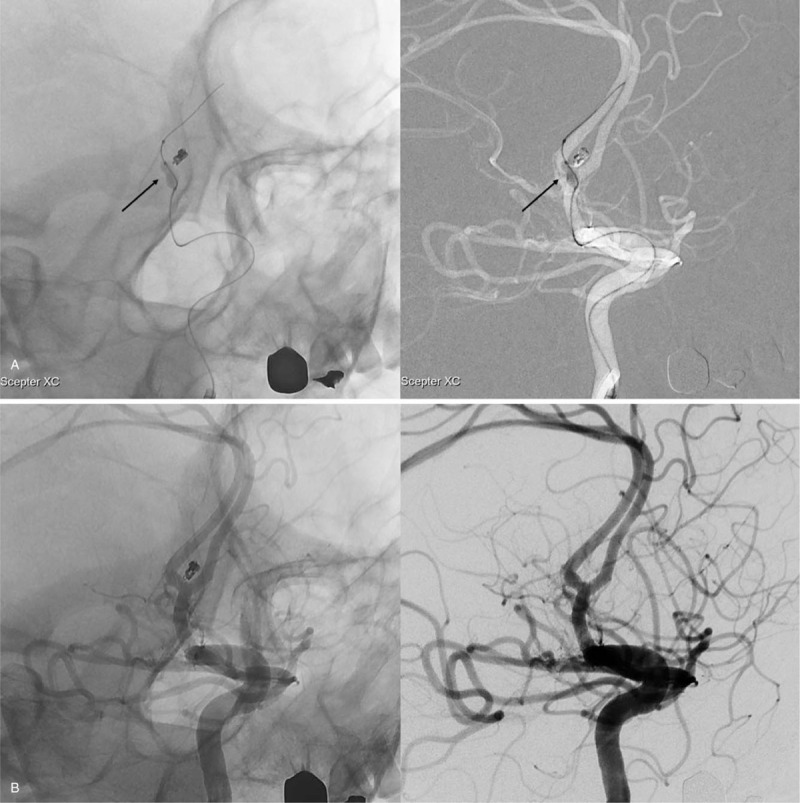
A rescue balloon-assisted remodeling procedure. (A) A Sceptor XC^TM^ balloon was inflated around the protruded coil and the aneurysm neck (black arrow). (B) The protruded coil was pushed back into the aneurysmal sac and thrombus disappeared.

## 3 Discussion

With an increase in the number of aneurysmal coiling procedures, the incidence of protrusion of the detachable coil into the parent artery during endovascular embolization of intracranial aneurysms is also increasing. Thus, it is considered a potentially critical intraprocedural complication.^[2,6]^ However, there are very few studies or reports on effective management of coil protrusion. In the absence of thrombus formation or occlusion of the parent artery around the protruded coil loops, coil protrusion may be treated conservatively with antiplatelet or anticoagulation therapy.^[9]^ However, as observed in the present case, coil protrusion into the parent artery may cause thrombus formation, which leads to occlusion of the parent artery or that of major branches of the distal arteries, consequently resulting in territory infarction with neurologic deficits. Therefore, in such situations, immediate intervention is absolutely necessary to avoid significant morbidity and mortality.

Various salvage methods for the management of protruded coils into the parent artery during endovascular coil embolization of intracranial aneurysms have been demonstrated. These include endovascular retrieval of the protruded coils, placement of stent devices, and microsurgical removal of the protruded coils.^[1,2,6]^ Generally, microsurgical removal is performed if failure of salvage methods using the endovascular approach is encountered.^[10]^ In the majority of the cases, microsurgical removal of the intravascular coils is associated with an extremely high risk of complications that outweigh the potential advantages.^[11]^ In the present case, since thrombus formation was already in progress, parent artery occlusion leading to territory infarction could have occurred during the time required to perform microsurgical removal of the coils. Stent placement on the protruded coils is an effective technique to fix the coils to the parent artery.^[6]^ However, stent placement may have a possible risk of stent delivery to the tortuous distal intracranial arteries beyond the protruded coils, as this procedure may disturb the deployed coils. Another possible complication of stent placement is a stent-induced thromboembolic event. It has been demonstrated that the incidence of thromboembolic events is high when stents are deployed in the absence of antiplatelet treatment.^[12]^ In the present case, the patient had not undergone pretreatment with antiplatelet medication. Moreover, even if endovascular retrieval of the protruded coils is the best strategy in case of a ruptured aneurysm, there are potential hazards such as embolic migration of the coils from the aneurysm to the distal intracranial arteries, vessel injuries resulting from relatively hard retrieval systems, and displacement of previously placed coils.^[13]^

Since the first description of balloon-assisted coil embolization of intracranial aneurysms,^[3,13]^ this technique has been used to prevent coil loop protrusion from the aneurysmal sac into the parent artery during coil insertion into the aneurysmal sac, especially in an aneurysm with wide neck.^[3,13]^ With temporary balloon inflation behind the neck of the aneurysm, the detachable coils are inserted into the aneurysmal sac, which provides the coil configuration within the confines of the aneurysm.^[3]^ On this technical assumption, we decided to reposition the protruded coil into the aneurysmal sac using balloon inflation (rescue balloon-assisted remodeling). Fortunately, the protruded coil was successfully repositioned into the aneurysmal sac with a rescue balloon-assisted remodeling technique. In the present case, protrusion of a small portion of the coil loop occurred during the removing of the microcatheter from the aneurysmal sac after detaching the last coil. In such cases, a rescue balloon-assisted remodeling can be considered a suitable technique for the management of coil protrusion with minimal possible complications compared to other salvage procedures.

## 4 Conclusions

We have described a rescue balloon-assisted remodeling technique to manage coil loop protrusion into the parent artery during endovascular coil embolization of an intracranial aneurysm. If the protruded coil loop is small, coil repositioning with balloon inflation should be preferred to other salvage procedures.

## Acknowledgments

We would like to thank the patient who consented to this report and Editage (www.editage.com) for English language editing.

## Author contributions

**Conceptualization:** Hak Cheol Ko.

**Visualization:** Hak Cheol Ko.

**Writing – original draft:** Hak Cheol Ko.

**Writing – review & editing:** Hak Cheol Ko.

## Supplementary Material

Supplemental Digital Content
